# Investigation of SiC Trench MOSFETs’ Reliability under Short-Circuit Conditions

**DOI:** 10.3390/ma15020598

**Published:** 2022-01-13

**Authors:** Yuan Zou, Jue Wang, Hongyi Xu, Hengyu Wang

**Affiliations:** 1 School of Information & Electrical Engineering, Zhejiang University City College, Hangzhou 310015, China; zouy@zju.edu.cn; 2 College of Electrical Engineering, Zhejiang University, Hangzhou 310027, China; xuhongyi@zju.edu.cn (H.X.); wanghengyu@zju.edu.cn (H.W.)

**Keywords:** SiC trench MOSFET, short-circuit failure mechanism, failure analysis

## Abstract

In this paper, the short-circuit robustness of 1200 V silicon carbide (SiC) trench MOSFETs with different gate structures has been investigated. The MOSFETs exhibited different failure modes under different DC bus voltages. For double trench SiC MOSFETs, failure modes are gate failure at lower dc bus voltages and thermal runaway at higher dc bus voltages, while failure modes for asymmetric trench SiC MOSFETs are soft failure and thermal runaway, respectively. The shortcircuit withstanding time (SCWT) of the asymmetric trench MOSFET is higher than that of the double trench MOSFETs. The thermal and mechanical stresses inside the devices during the short-circuit tests have been simulated to probe into the failure mechanisms and reveal the impact of the device structures on the device reliability. Finally, post-failure analysis has been carried out to verify the root causes of the device failure.

## 1. Introduction

Over the past few decades, wide-bandgap semiconductors like SiC have become more attractive compared to traditional silicon devices due to their high breakdown field, high thermal conductivity, and wide bandgap [1,2]. By now, iC devices such as Schottky diodes have been rapidly developed and are widely used commercially [3,4]. However, the short-circuit performance of SiC MOSFETs is still poor compared to Si-IGBTs. Due to their smaller area and higher power density, SiC MOSFETs have higher junction temperatures than Si-IGBTs and tend to suffer from thermal runaway [5,6,7,8]. In addition, the poor interface state problem of SiC MOSFETs can also lead to gate reliability issues, causing the device gate failure [9,10].

As SiC material growth and device fabrication technologies have evolved, the structure of SiC MOSFETs has become increasingly sophisticated. Today, various commercial SiC MOSFETs with planar and trench gate structures from different manufacturers are available [11,12]. Compared to SiC planar gate MOSFETs, trench gate MOSFETs have higher power density and lower conduction resistance [13]. Although SiC trench MOSFETs have many advantages, their reliability needs further research due to the defects introduced during manufacturing process [14]. First, the inhomogeneity of oxidation during gate formation makes the oxide thickness at the sidewall and trench bottom inconsistent. The oxidation inconsistency increases the SiO${}_{2}$/SiC surface roughness, and leads to local electric field concentration at the rough point. Hence, more charges are injected into the gate oxide, increases the charge through the gate oxide, shortening the time to dielectric breakdown [15,16]. Otherwise, threading dislocations (threading screw dislocations (TSDs) and threading edge dislocations (TEDs)) can cause significant leakage points in the device, which can severely degrade the performance of the SiC device [17,18]. The second issue is the infamous interface state problem. In addition, SiC devices tend to be operated under high voltage conditions, which makes the gate oxide layer bear a high electric field. In response, different shielding methods have been proposed, such as double trench MOSFETs (DT-MOSFETs) and asymmetric trench MOSFETs (AT-MOSFETs) [19,20]. The short-circuit failure modes of DT-MOSFETs have been reported in some detail [21,22], while the short-circuit reliability of AT-MOSFETs has been less studied. A comparison of the two devices’ short-circuit reliability has been reported [23], but it focuses more on the safe operating region and failure prediction regarding the device failure mode. The difference in the internal thermal and mechanical stress during the short-circuit process related to the difference in device structures has not been addressed.

In this paper, the short-circuit ruggedness of two 1200 V SiC trench MOSFETs with different gate oxide shielding methods (double-trench and asymmetric-trench) has been investigated. The device characteristics were recorded and analyzed. The maximum short-circuit withstand time (SCWT) of the devices have been measured and compared. To delve into the failure mechanism, the short-circuit process of the devices has been simulated by using a two-dimensional finite element numerical simulation tool. The distribution of currents, lattice temperatures, and mechanical stresses inside the devices were investigated in detail. Finally, the failure was determined by using a failure analysis tool.

## 2. Device Structure and Experiment Setup

### 2.1. Device Structure

**Figure 1:**
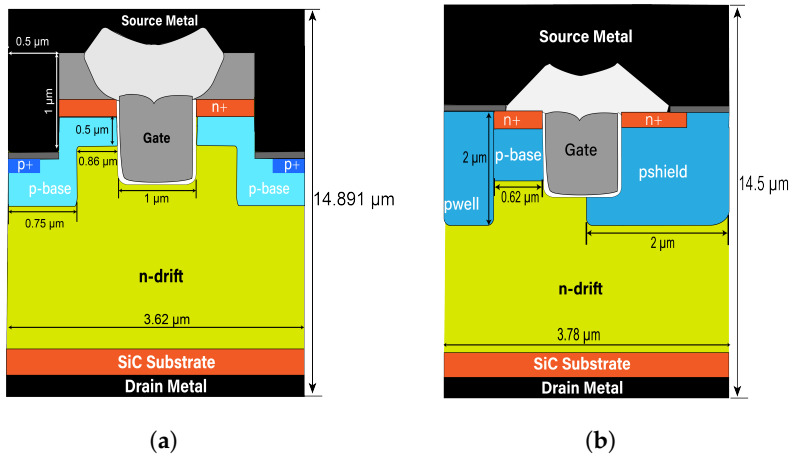
Cross-section images of the two trench MOSFETs. (**a**) DT-MOSFET. (**b**) AT-MOSFET.

**Figure 2:**
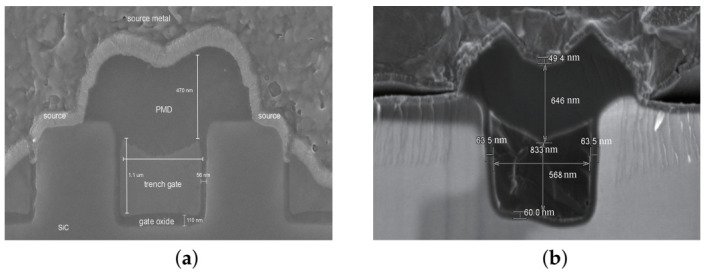
Cross-section images of the two trench MOSFETs by FIB and SEM. (**a**) DT-MOSFET. (**b**) AT-MOSFET.

The short-circuit reliability of two 1200 V SiC commercial power trench MOSFETs manufactured by Rohm and Infineon, respectively, have been chosen as the devices under test (DUTs) [24,25]. The main electrical parameters of the devices have been listed in Table 1. Figure 1 shows the cell structure of two devices. The structure parameters of the devices have been obtained by SEM and FIB, as shown in Figure 2. The doping concentrations of the device have been obtained by fitting the device characteristics (transfer curve, output curve, breakdown voltage, etc.) by the numerical simulation.

### 2.2. Experiment Setup

**Figure 3:**
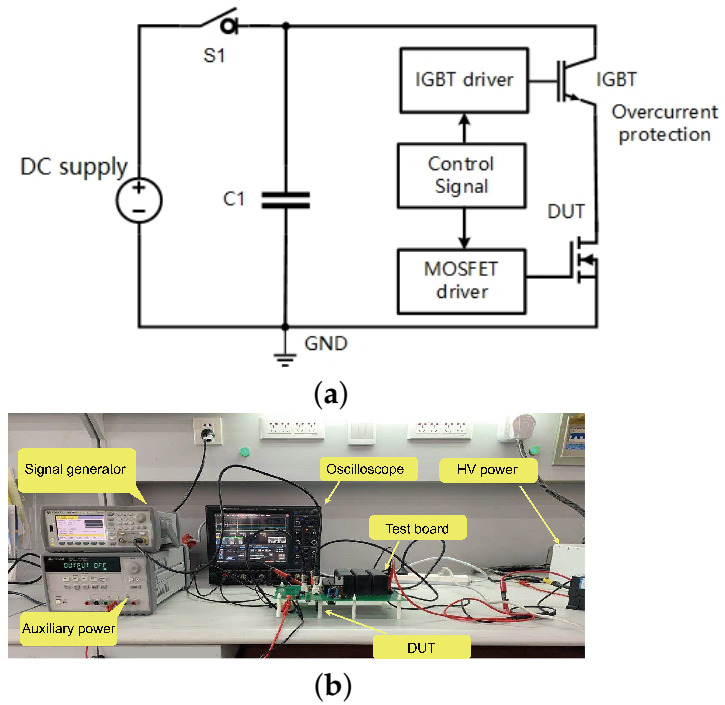
Circuit schematic diagram: (**a**) Simplified schematic of the short-circuit test. (**b**) Photograph of the test platform.

Figure 3 shows the short-circuit test circuit schematic diagram and the photograph of the test platform. To provide sufficient energy during the short-circuit, C1 consisted of six 50 μF/1200 V capacitors [26]. In order to avoid device catastrophic failure, an IGBT [27] was employed as the solid-state circuit breaker. Initially, both the IGBT and the DUT were kept off. At first, S1 was turned on and the capacitor C1 was charged to a high voltage via a DC power supply. Afterwards, S1 was turned off and the short-circuit stress was applied to the DUT. The short-circuit pulse width was varied by controlling the gate signals of the IGBT and the DUT. The short-circuit capability of the device can be quantified by the short-circuit withstand time (SCWT), reflecting the maximum short-circuit time that the device can tolerate. After every single test, the device was cooled down to room temperature before the next test started to prevent the heat accumulation inside the devices.

## 3. Experiment Results

### 3.1. DT-MOSFET

**Figure 4:**
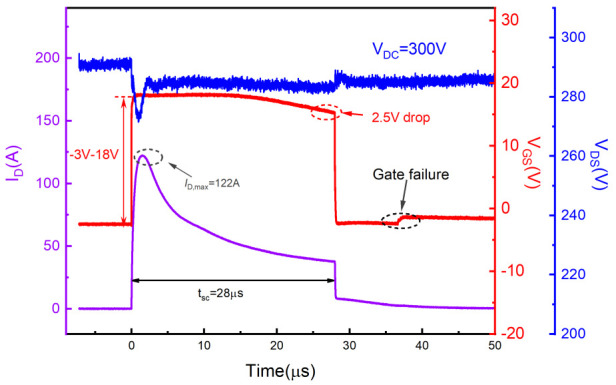
Short-circuit failure waveforms for the SiC DT-MOSFETs at 300 V DC bus voltage.

Figure 4 shows typical experimental short-circuit waveforms of SiC DT-MOSFETs with V${}_{DC}$ = 300 V and V${}_{GS}$ = 18 V/−3 V. When the device is turned on, internal parasitic parameters of the device and the test board cause a brief overshoot on V${}_{GS}$ and V${}_{DS}$. However, this overshoot does not affect the following short-circuit process [28]. The short circuit pulse width $t_{sc}$ was gradually increased to 28 μs until the device reached the failure point, accompanied by a peak current value of 125 A and a V${}_{GS}$ drop of 2.5 V. The anomaly only showed on the gate voltage waveform, manifested as a sudden increase of V${}_{GS}$ (from −3 V to 0 V) after the device has been turned off for 7 μs, whereby the drain-source voltage still maintained to DC bus voltage. This means that the gate and source terminals are shorted, while the drain-source body diode still has blocking capability. The measured waveforms indicate the gate failure mode [10,29,30]. The same result was demonstrated in the subsequent electrical inspection of the three terminals, as shown in Table 2.

**Figure 5:**
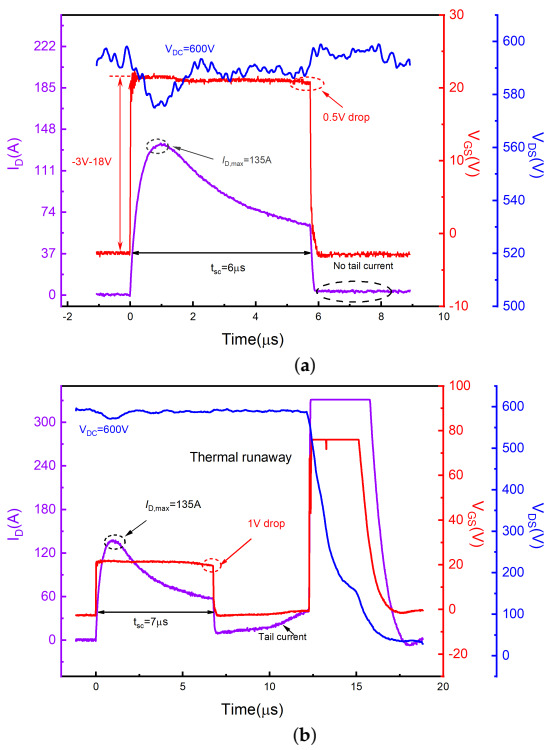
Short-circuit waveforms for the SiC DT-MOSFETs at 600 V DC bus voltage. (**a**) Last waveforms before failure and (**b**) failure.

Figure 5 shows the short-circuit waveforms of SiC DT-MOSFETs measured before the failure (a) and at the failure (b), when increasing the bus voltage to 600 V. Higher DC bus voltage leads to higher power dissipation, causing the SCWT to decrease to 7 μs. When the device fails, the peak current value is about 135 A, accompanied by the V${}_{GS}$ drop of about 1 V. It can be seen from Figure 5b that a significant trail current appears after the device is turned off, climbing up to 39 A. High junction temperature caused by high power consumption can significantly increase the carrier density. Therefore, the device cannot be completely shut down at the end of short-circuit operation [31]. The hole current is the main cause of tail currents. Due to the existence of the tail current, more heat is generated, forming a positive feedback. If the tail current exceeds the threshold current to trigger the parasitic BJT, the hole density and the junction temperature will increase further and finally lead to thermal runaway [32]. As listed in Table 2, the resistance between the three terminals (R${}_{GS}$, R${}_{GD}$, and R${}_{DS}$) after the short-circuit test became quite low, revealing that all electrodes were shorted.

### 3.2. AT-MOSFET

**Figure 6:**
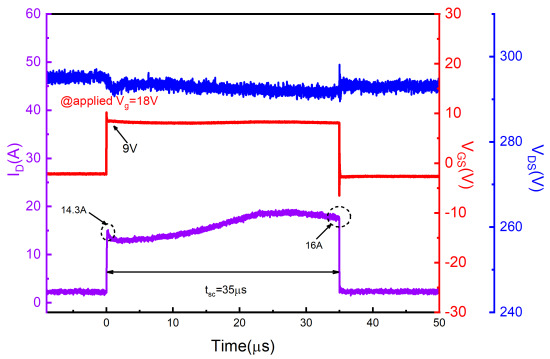
Short-circuit failure waveforms for the SiC AT-MOSFETs at 300 V DC bus voltage.

Figure 6 shows typical experimental short-circuit waveforms of SiC AT-MOSFETs at 300 V DC bus voltages, and gate bias was set as V${}_{GS}$ = 18 V/−3 V. Under the same conditions, the SiC AT-MOSFET shows better short-circuit performance than the DT-MOSFET (35 μs for AT-MOSFET, 28 μs for DT-MOSFET). However, the longer short-circuit time leads to more serious device damage. After withstanding a 35 μs short-circuit pulse, the device is no longer able to operate normally. First, the short-circuit current drops to a dozen amps and shows an abnormal upward trend. In addition, the gate voltage is 9 V/−2 V even external 18 V/−3 V is applied. This indicates that a leakage path is formed between the gate and source, but they are not completely shorted, which is referred to as the soft failure. Devices have been previously reported to fail at low bus voltages due to gate-source SiO${}_{2}$ rupture [33]. Therefore, it can be inferred that the gradual accumulation of dielectric layer damage in AT-MOSFETs under prolonged short-circuit stress may be the root cause of soft failure. The device body diode is still able to carry 300 V. The results of the subsequent electrical inspection of the three terminals are shown in Table 3.

**Figure 7:**
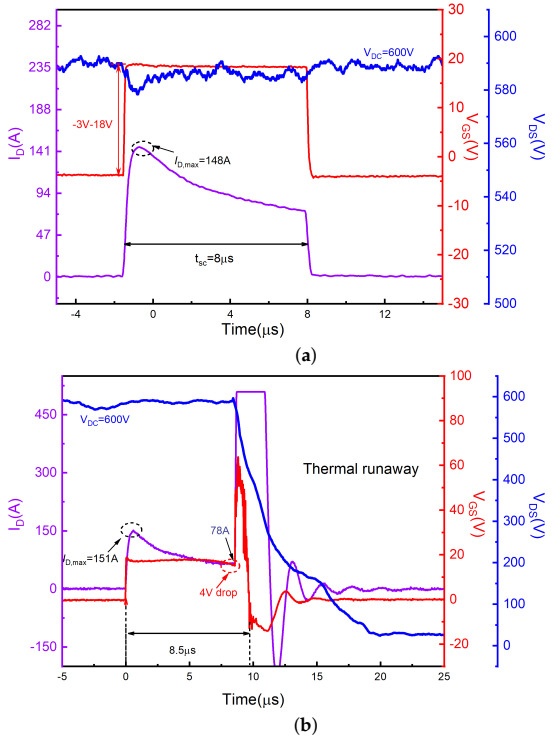
Short-circuit waveforms for the SiC AT-MOSFETs at 600 V DC bus voltage. (**a**) Last waveforms measured before failure and (**b**) at failure.

Figure 7 shows short-circuit waveforms of SiC AT-MOSFETs at 600 V DC bus voltages, and gate bias was set as V${}_{GS}$ = 18 V/−3 V. At higher bus voltage, the device is subjected to higher power dissipation and the AT-MOSFET exhibits a thermal runaway mode after a 8.5 μs SC pulse. The performance is slightly better than that of DT-MOSFETs, but it is somewhat different from the thermal runaway mode of DT-MOSFETs. First, the thermal runaway of the AT-MOSFETs does not occur after the device is turned off, but during the period when the short-circuit stress is applied. A comparison with the last waveforms measured before failure shows that the current increases during the short-circuit pulse. For example, the drain current increased from 66 A to 78 A at 7 μs. This indicates that a trailing current has occurred during the short-circuit pulse. As the junction temperature increases further, the current value is sufficient to trigger the parasitic BJT before the device shuts down. Compared to the DT-MOSFETs, the higher power level of the AT-MOSFET results in a higher peak current than the DT-MOSFETs, making the junction temperature rise faster, thus causing the thermal runaway mode to be triggered before the device is turned off. The second point is that the gate-source voltage of the AT-MOSFETs exhibites anomalies during the short-circuit pulse. There is a gate voltage drop of about 4 V near the point of failure, indicating that a high leakage current is flowing across the gate resistance. This indicates that the gate degradation occurs also. However, the junction temperature rises rapidly due to the higher bus voltage, so that there is not enough time for the gate to be damaged seriously before the thermal runaway occurs. The electrical characteristics in Table 3 also shows that all three terminals of the device are shorted together.

**Figure 8:**
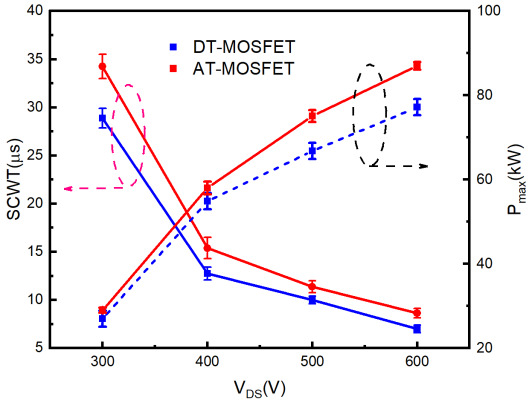
SCWT and Extracted $P_{\max}$ comparison of different DUTs under different conditions.

The results of SCWT and extracted $P_{\max}$ comparison between DUTs are shown in Figure 8. The maximum power dissipated in the short-circuit test is higher because the AT-MOSFETs has a higher current rating than the DT-MOSFETs. However, the survival time of DT-MOSFETs at different bus voltages are shorter than that of AT-MOSFETs, which needs further study.

## 4. Failure Mechanism

### 4.1. Finite Element Numerical Simulation

To investigate the internal behavior of SiC MOSFETs during short-circuit stress and to obtain deep insight into the failure mechanisms, Sentaurus TCAD software was employed to evaluate the electrical-thermal-mechanical stress distribution of these two trench gate structures at room temperature (300 K). The device structure parameters used in the simulations are shown in Table 4.

Some critical dimension parameters are marked in Figure 1.

**Figure 9:**
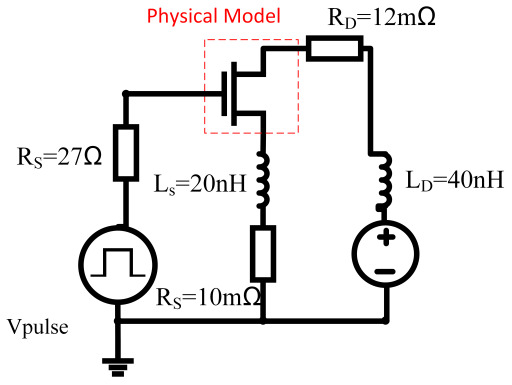
The mixed-mode schematic in Sentaurus TCAD.

The mixed-mode transient simulations (Sentaurus TCAD) have been performed to study the short-circuit reliability. The test circuit schematic is shown in Figure 9. In the simulation, the characteristics of the MOSFET were solved by finite element numeric analysis, while other components were modeled by Spice electric models. Additional passive elements were included to consider the actual parasitic effects in the circuit. Specifically, the stray inductance and parasitic resistance at the MOSFET source terminal L${}_{S}$, R${}_{S}$) affect the di/dt in the conduction mode, and the stray inductance L${}_{D}$ at the drain terminal is used to simulate voltage spikes during the switching transients.The values of these parasitic parameters are obtained by simulation fitting the actual switching curve, and the temperature of the substrate was set to be 300 K. Considering the self-heating effect of the device, the junction temperature was solved and updated during the simulations. The short-circuit pulse width is set to be 12 μs. At the end of the pulse, the current density and temperature distribution inside the device were extracted to investigate the failure mechanism. Then, the simulated temperature distribution from the electro-thermal simulation was extracted and imported into Sentaurus interconnect [34]. Using the temperature information, the mechanical stress inside the device was calculated.

**Figure 10:**
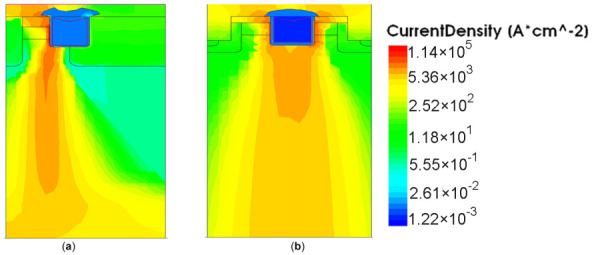
Conduction current density distribution in the device (when the peak junction temperature occurs): (**a**) DT-MOSFET(**b**) AT-MOSFET.

The current density distribution of the DT-MOSFETs and AT-MOSFETs are plotted in Figure 10. As can be seen from Figure 10, because the AT-MOSFET in forward conduction mode only uses half of the channel, the device’s on-state current is more concentrated on one side, while in the DT-MOSFET’s the current is uniformly distributed on both sides. The current distribution between the two structures also results in a difference in temperature distribution. As in the AT-MOSFET a built-in JFET is formed around the trench gate structure of the deep P-Shield, the current density in this JFET region can be effectively reduced. In turn, it causes the current to spread deeper into the device internal region. Since the melting temperature of the electrode metal is much lower than that of SiC, pushing the current away from the metal/SiC interface enables the device to withstand longer short-circuit stress. Therefore, the SCWT of the AT-MOS is still higher than that of the DT-MOS. However, the channel of this JFET is directly controlled by V${}_{DS}$. Therefore, when V${}_{DS}$ increases, the single-side short-circuit current density at the junction near the P${}_{base}$ and N${}_{drift}$ increases rapidly, and local self-heating occurs, leading to the triggering of the parasitic transistor and hence early thermal runaway.

**Figure 11:**
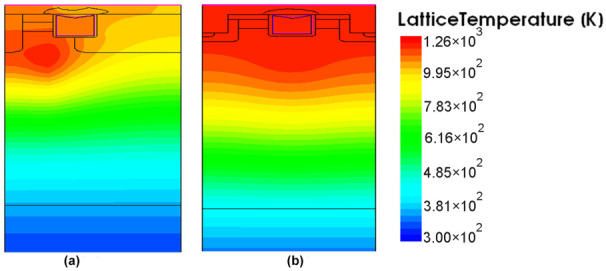
Lattice temperature distribution in the device (when the peak junction temperature occurs): (**a**) DT-MOSFET (**b**) AT-MOSFET.

The temperature distribution of the DT-MOSFETs and AT-MOSFETs are shown in Figure 11. For the DT-MOSFET, the current distribution is also symmetrical due to its symmetrical structure, and the temperature drops uniformly from source to drain. The simulated temperatures in the SiC and Al regions are 1371 K and 1382 K, respectively, which already exceed the melting point of Aluminum. For the AT-MOSFET, the temperature is mainly concentrated on one side due to the deep p-region surrounding the gate. Its highest temperature is concentrated in the body region. The aluminum metal near the conduction side is subjectedto a higher temperature than the other side. Likewise, the temperature of the aluminum metal near the on-state side exceeds the melting point of aluminum. Therefore, the melting of the upper aluminum metal may also occur during the short-circuit operation.

**Figure 12:**
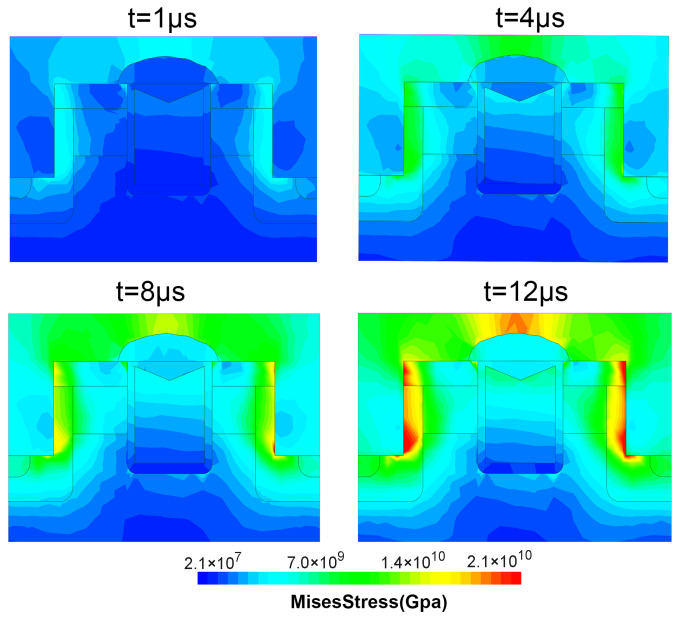
The von Mises stress distribution in the DT-MOSFET during SC condition.

**Figure 13:**
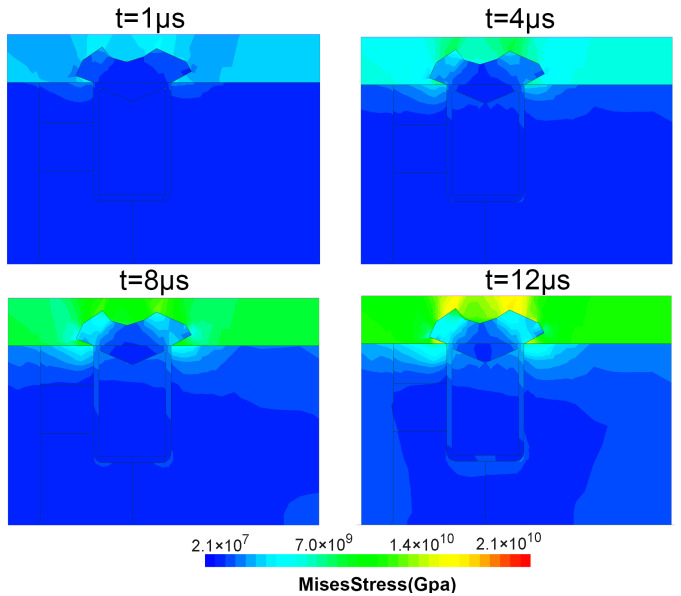
The von Mises stress distribution in the AT-MOSFET during SC condition.

Table 5 shows the melting points and thermal expansion coefficients of the materials used in the MOSFET manufacturing process. Since the thermal expansion of aluminum is more significant than that of SiC and the insulators, large mechanical stresses may be generated at the SiO${}_{2}$ layer and the Al/SiC interface, which may lead to cracks in these regions [35]. As the junction temperature of the device increases during the short-circuit stress, the temperature gradient increases and the thermal stress inside also increases. As shown in Figure 12, with the increase of short-circuit pulse width, the simulated stress is concentrated in the gate insulation layer and near the source trenchand increases gradually versus time. Finally, the stress in these regions exceeds the materials’ strength, and cracks occur. It is possible that this is the root cause of the gate-source short. In addition, the mechanical stress in the AT-MOSFET is simulated and plotted in Figure 13. Due to the lower temperature distribution in the metal part than in the DT-MOSFET, the mechanical stress in the metal above SiC regions is lower than that of the DT-MOSFETs.

### 4.2. Post-Failure Analysis

**Figure 14:**
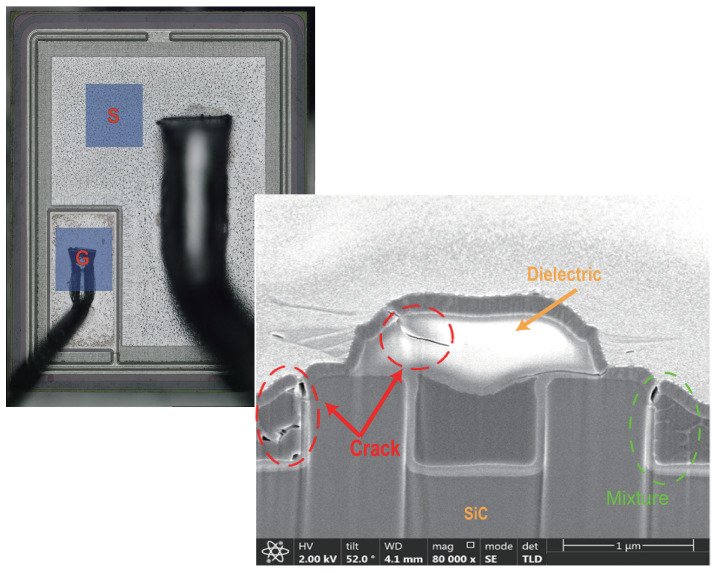
Cross-sectional SEM image of failed DT-MOSFET cell at 300 V DC voltage. The red dotted circles identify the cracks.

**Figure 15:**
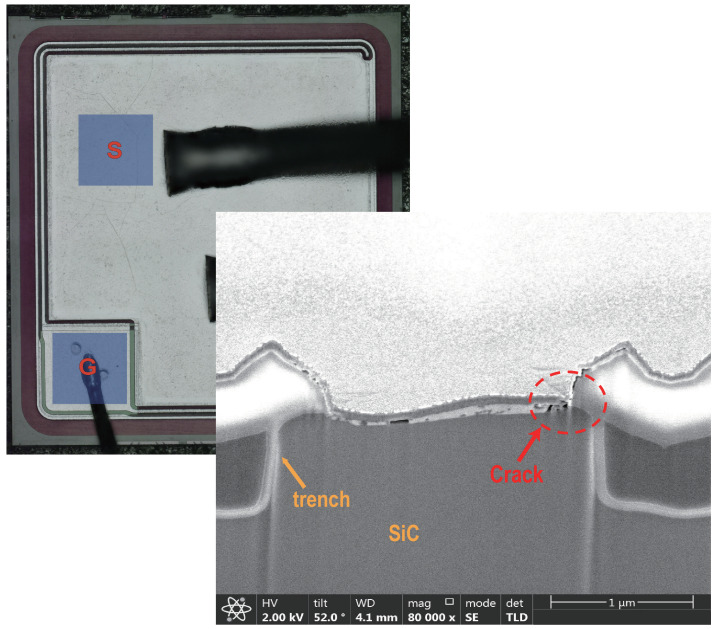
Cross-sectional SEM image of failed AT-MOSFET cell at 300 V DC voltage. The red dotted circles identify the cracks.

To further investigate the failure mechanisms of the devices and to verify the conclusions drawn by performing device simulations, failure analysis has been carried out on the failed devices. Figure 14 and Figure 15 show the failed DT-MOSFETs and AT-MOSFETs at 300 V bus voltage, respectively. The DT-MOSFET failed due to gate failure and the AT-MOSFET failed due to soft failure. The bonding wires and joints remain intact and no visible damage can be observed on the chip surface. By using focused ion beam (FIB) dicing (based on emission microscopy analysis), the locations of the cracks inside the devices have been identified.

**Figure 16:**
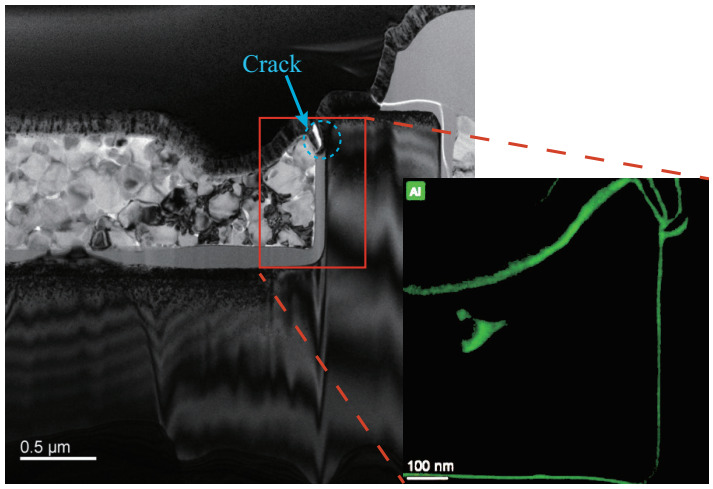
Atom spectrum analysis after failure. The green part represents the distribution of aluminium.

For the DT-MOSFETs, the cracks appear at the corner of the source trench and the pre-Metal dielectric of the gate. In addition, a suspected metal mixture was found in the trench corners. Therefore, the energy- dispersive X-ray spectroscopy (EDX) was employed to analyze the composition of the metal particles, (Figure 16). It was identified as the elemental Aluminum, indicating that the melting of aluminum occurred during the short-circuit operation. Due to the thermal expansion of the materials with the rising temperature, the stress in these regions exceeded the limit of the material and cracks appearred. Next, the melted aluminum flowed into the cracks. Since it took time for Aluminum to fill the cracks, the gate failure occurred within a few microseconds after the device is turned off. For AT-MOSFETs, the damage is less than observed in DT-MOSFETs. The failure point appears in the metal above the channel, while the pre-Metal dielectric of the gate remains intact. Therefore, the AT-MOSFET showed a soft failure, with a large amount of leakage gate current, but was still able to support a part of the applied voltage.

**Figure 17:**
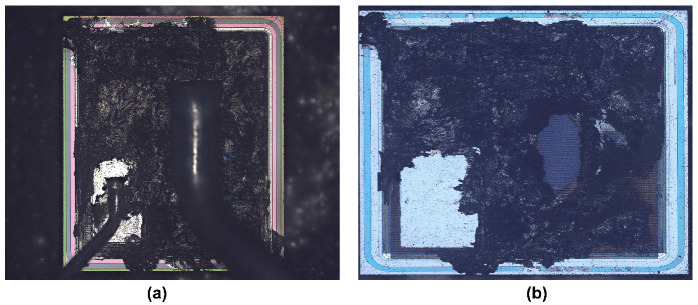
Images of failed DT-MOSFET at 600 V DC voltage. (**a**) Surface. (**b**) Substrate.

**Figure 18:**
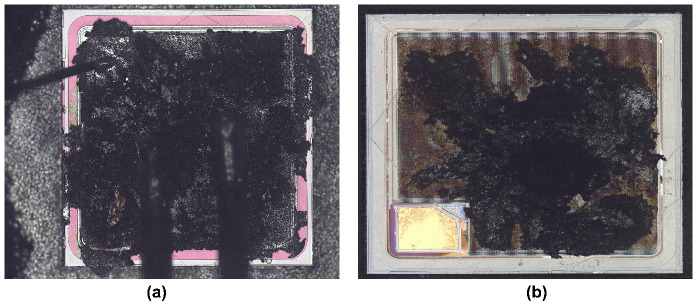
Images of failed AT-MOSFET at 600 V DC voltage. (**a**) Surface. (**b**) Substrate.

Figure 17 and Figure 18 show the failed DT-MOSFETs and AT-MOSFETs after the 600 V test, respectively. At 600 V, the entire surface has been burned due to the triggering of the parasitic transistors. To further observe the device burn-ins, the device was dissected to the substrate layer. It was found that the burned area was mainly concentrated near the source bonding wires, verifying that the high temperature caused by the high current induced the thermal runaway and catastrophic failure of the device. In addition, the burned area of the AT-MOSFET on the substrate is smaller compared to that of the DT-MOSFET. Since only one side of the AT-MOSFET conducts when it turns on, resulting in localized overheating during the short circuit, as a result, the damage point is more concentrated.

## 5. Conclusions

The short-circuit performance of 1200 V SiC trench MOSFETs with asymmetric and double-trench shielding structures has been investigated. Three SiC MOSFET failure mechanisms have been identified: thermal runaway, gate failure, and soft failure modes. The double trench MOSFETs failed with thermal runaway and gate failure modes. For asymmetric trench MOSFETs, the failure modes were thermal runaway and soft failure. The AT-MOSFETs exhibit better short-circuit performance under the same conditions. The numerical simulation results reveal that the deep P-region around the gate in the AT-MOSFETs can effectively limit the short-circuit current, due to the JFET region formed with the adjacent Pbase region. Thus, the current can be distributed more evenly into the device, allowing the maximum temperature to penetrate deeper into the body region, thus preventing the relatively fragile metal and dielectric layers on the surface from exposure to excessive heat. Further numerical simulations have indicated that the mechanical stress is the root cause of gate failure. The post-failure analysis has located cracks in the insulation layer and the source metal. In addition, EDX elemental analysis confirmed the presence of molten aluminum into the cracks, proving the credibility of the simulation results.

## Figures and Tables

**Table 1:** Device rated parameters.

Rated Parameters	Rohm (DT-MOSFET)	Infineon (AT-MOSFET)
$V_{\mathrm{DS}}$	1200 V	1200 V
$R_{\mathrm{DS}(\mathrm{on})}\left(\mathrm{Typ}.\right)$	160 mΩ	90 mΩ
$I_{D}$	17 A	26 A
Power dissipation	103 W	115 W
Drive Voltage	0 V/18 V	0 V/15–18 V
Junction temperature	175 ${}^{\circ }\;C$	175 ${}^{\circ }\;C$
Active area	3.1378 mm${}^{2}$	3.0751 mm${}^{2}$
Package	TO-247N	PG-TO247-3
Orderable Part Number	SCT3160KLGC11	IMW120R090M1HXKSA1

**Table 2:** DT-MOSFET: Measured resistances between electrodes before and after tests.

Device State	R${}_{\mathrm{GS}}\left(\Omega \right)$	R${}_{\mathrm{DS}}\left(\Omega \right)$	R${}_{\mathrm{GD}}\left(\Omega \right)$
fresh device	>100 M	1.529 M	>100 M
V${}_{DS}$ at 300 V	8.6	>100 M	84 k
V${}_{DS}$ at 600 V	3.36	3.05	2.36

**Table 3:** AT-MOSFET: Measured resistances between electrodes before and after tests.

Device State	R${}_{\mathrm{GS}}\left(\Omega \right)$	R${}_{\mathrm{DS}}\left(\Omega \right)$	R${}_{\mathrm{GD}}\left(\Omega \right)$
fresh device	>100 M	1.529 M	>100 M
V${}_{DS}$ at 300 V	6.86	0.128 M	>100 M
V${}_{DS}$ at 600 V	3.36	3.12	2.54

**Table 4:** Device structure parameters.

Simulation Parameters	DT-MOSFET	AT-MOSFET
Substrate layer’s Doping (cm${}^{-3}$)	$1\times 10^{19}$	$1\times 10^{19}$
Epitaxial layer’s thickness (μm)	10.5	10.2
Drift-region doping (cm${}^{-3}$)	$5\times 10^{15}$	$1\times 10^{16}$
P-base doping (cm${}^{-3}$)	$2\times 10^{16}$	$3\times 10^{16}$
N+ source doping (cm${}^{-3}$)	$1\times 10^{18}$	$1\times 10^{18}$
Oxide sidewall’s thickness (nm)	56	63.5
Oxide bottom’s thickness (nm)	110	60

**Table 5:** Melting point and thermal expansion coefficient of materials.

Materials	Melting Point (${}^{\circ }$C)	CTE ($10^{-6}/$K)	Young’s Modulus (GPa)
SiO${}_{2}$	1650	0.5	75
Poly-silicon	1410	2.9	169
SiC	2700	6.58	500
Al	660	23.2	68

## Data Availability

The data presented in this study are available upon request from the corresponding author.
